# Metformin and lactic acidosis during shock: just the tip of the iceberg?

**DOI:** 10.1186/s13054-016-1333-2

**Published:** 2016-06-01

**Authors:** Rene A. Posma, Anthony R. Absalom, Daan J. Touw, Iwan C. C. van der Horst, Maarten W. N. Nijsten

**Affiliations:** Department of Critical Care, University Medical Center Groningen, University of Groningen, Groningen, The Netherlands; Department of Anesthesiology, University Medical Center Groningen, University of Groningen, Groningen, The Netherlands; Department of Clinical Pharmacy and Pharmacology, University Medical Center Groningen, University of Groningen, Groningen, The Netherlands

## Letter

Doenyas-Barak et al. concluded recently in *Critical Care* that with similar high lactate levels (>10 mmol/L) the prognosis of metformin users compared with non-users was favorable during admission to the emergency department with septic shock (in-hospital mortality, 57 % versus 88 %, *P* < 0.001) [1]. This observation and similar findings reported previously are suggestive of a role for metformin as a lactate generation amplifier [2, 3]. However, in this study, causality cannot be proven, since without metformin concentration measurements a dose-response analysis is not possible.

Despite the dramatic clinical presentation of metformin-associated lactic acidosis (MALA), this study also underlines the fact that its prognosis is better than that of similarly severe lactic acidosis of other origins [1, 4]. With an incidence of approximately 5 cases per 100,000 patient-years, MALA is considered an exceedingly rare complication of chronic metformin therapy [5]. Metformin is excreted unchanged by the kidneys, predisposing patients with renal insufficiency to the development of MALA. Several authors suggest that the inclusion of renal impairment as a contraindication for metformin use should be reconsidered, and this may increase the number of patients at risk for developing MALA [5].

The findings reported in this study shed new light on our current understanding of the impact of metformin on lactate production. Given the large difference in mortality between the two groups, it can be concluded that the prognostic value of lactate is largely altered by metformin use in patients presenting with shock. Evidently, a relatively small sample size of patients (44 metformin users and 118 non-users) is sufficient to observe differences of such a magnitude in survival. Although the effect may not be as pronounced as in the present study, we believe that metformin may also elevate lactate levels in patients presenting with less severe lactic acidosis [1, 2]. Therefore, a study comparing the prognosis of critically ill metformin users with non-users in a large matched cohort based on increasing lactate levels seems useful.

Because the effect of metformin on lactate levels in such a population may not be immediately obvious, we suspect that hyperlactatemia partly induced by metformin is often not considered by physicians caring for patients presenting with critical conditions. This study might therefore have identified only the tip of the iceberg regarding the effect of metformin on lactate levels. For clinicians to be able to identify and treat MALA in a more timely manner, lactate levels should always be measured in the emergency department or even in the prehospital setting.

## Abbreviations

CKD: Chronic kidney disease
MALA: Metformin-associated lactic acidosis
