# Supplementary material for: Metformin and lactic acidosis during shock: just the tip of the iceberg?
Source: Crit Care. 2016 Jun 1;20:158. doi: 10.1186/s13054-016-1333-2 (PMC4888659; doi:10.1186/s13054-016-1333-2)
Supplement: Related texts

# Reply: Authors’ response

**Authors:** Keren Doenyas-Barak, Shai Efrati

The main finding of our study was that patients under metformin treatment with severe lactic acidosis and septic shock have better prognosis than patients without metformin treatment [1]. The difference can be attributed to metformin accumulation, representing MALA, and not the severity of the sepsis. In such cases, the blood level of metformin would be expected to be high. However, the difference between the groups may also represent a metformin-related protective effect, in which case the blood level of metformin would not necessarily be high. Thus, lack of knowledge regarding circulating metformin levels is indeed a limitation of the present study. Nonetheless, it is clear that severe lactic acidosis under metformin treatment should be regarded as a different entity. Thus, we agree that subclinical MALA may be uncovered (or exposed) in some patients if larger populations are evaluated and if blood lactate is measured in all metformin users.

Posma et al. express concern that the low incidence of MALA may change if renal failure as a contraindication is reconsidered [6]. However, in the present cohort, acute rather than chronic renal failure was associated with MALA [1]. Metformin accumulates in patients with renal failure, and its plasma level was found to be in good correlation with renal function [7]. Thus, low-dose metformin treatment in patients with stable chronic renal failure may be safe. However, acute renal failure may pose the risk of acute metformin accumulation and lactic acidosis [8]. Thus, the risk for acute renal failure should always be considered before prescribing metformin.
